# Electrophysiological evidence for voltage-gated calcium channel 2 (Ca_v_2) modulation of mechano- and thermosensitive spinal neuronal responses in a rat model of osteoarthritis

**DOI:** 10.1016/j.neuroscience.2015.07.073

**Published:** 2015-10-01

**Authors:** W. Rahman, R. Patel, A.H. Dickenson

**Affiliations:** Department of Neuroscience, Physiology and Pharmacology, University College London, Gower Street, London WC1E 6BT, UK

**Keywords:** ANOVA, analysis of variance, DMSO, dimethylsulfoxide, MIA, monosodium iodoacetate, OA, osteoarthritis, PWT, paw withdrawal threshold, RM, repeated measures, TROX-1, N-triazole oxindole, vF, von Frey filament, VGCCs, voltage-gated calcium channels, WDR, wide dynamic range, pain, osteoarthritis, dorsal horn, voltage-gated calcium channel, *in vivo* electrophysiology

## Abstract

Osteoarthritis (OA) remains one of the greatest healthcare burdens in western society, with chronic debilitating pain-dominating clinical presentation yet therapeutic strategies are inadequate in many patients. Development of better analgesics is contingent on improved understanding of the molecular mechanisms mediating OA pain. Voltage-gated calcium channels 2.2 (Ca_v_2.2) play a critical role in spinal nociceptive transmission, therefore blocking Ca_v_2.2 activity represents an attractive opportunity for OA pain treatment, but the only available licensed Ca_v_2.2 antagonist ziconitide (PrilatTM) is of limited use. TROX-1 is an orally available, use dependent and state-selective Ca_v_2 antagonist, exerting its analgesic effect primarily via Ca_v_2.2 blockade, with an improved therapeutic window compared with ziconitide. Using a rat model of monosodium iodoacetate (MIA), 2 mg, induced OA we used *in vivo* electrophysiology to assess the effects of spinal or systemic administration of TROX-1 on the evoked activity of wide dynamic range spinal dorsal horn neurons in response to electrical, natural mechanical (dynamic brush and von Frey 2, 8, 26 and 6 g) and thermal (40, 45 and 45 °C) stimuli applied to the peripheral receptive field.

MIA injection into the knee joint resulted in mechanical hypersensitivity of the ipsilateral hind paw and weight-bearing asymmetry. Spinal administration of TROX-1 (0.1 and 1 μg/50 μl) produced a significant dose-related inhibition of dynamic brush, mechanical (von Frey filament (vF) 8, 26 and 60 g) and noxious thermal-(45 and 48 °C) evoked neuronal responses in MIA rats only. Systemic administration of TROX-1 produced a significant inhibition of the mechanical-(vF 8, 26 and 60 g) evoked neuronal responses in MIA rats. TROX-1 did not produce any significant effect on any neuronal measure in Sham controls. Our *in vivo* electrophysiological results demonstrate a pathological state-dependent effect of TROX-1, which suggests an increased functional role of Ca_v_2, likely Ca_v_2.2, channels in mediating OA pain.

## Highlights

• MIA-dependent antinociceptive effect of TROX-1 on neuronal activity.
• Alterations in Ca_v_2.2 channel function contribute to osteoarthritic (OA) pain.
• Blocking Ca_v_2.2 channels has therapeutic potential for treating OA pain.

## Introduction

Osteoarthritis (OA) is the most common form of joint disease, has a steadily rising prevalence due to an increasingly elderly and obese society, and represents one of the biggest contributors to the socioeconomic healthcare burden in the western world (Reginster, 2002). It is characterized by loss of articular cartilage, subchondral bone remodeling and inflammation and swelling of the joint. Perhaps the most defining feature of clinical OA is chronic debilitating joint pain. This can range from mild (dull aches) to severe (sharp stabbing pain) in the same patient, with consequent co-morbidities (mood and sleep problems) and decreased quality of life (Murphy et al., 2011). This would suggest abnormalities of peripheral and central processing of pain. Without any pharmacological disease-modifying therapies currently in use, treatment is predominantly analgesic: paracetamol forms the current first line, followed by NSAIDs, opioids and steroids in line with disease progression and the severity of pain. However these medicines are inadequate for many OA patients due to limited analgesic efficacy and safety issues, especially with prolonged use. This significant unmet clinical burden necessitates a greater understanding of the mechanisms that initiate and maintain OA pain in order to develop alternative, more effective analgesics.

Voltage-gated calcium channels (VGCCs) on nociceptors play an important role in nociceptive signaling; they are critical for neurotransmitter release, the regulation of neuronal excitability and intracellular changes (Lee, 2013). Studies have implicated an increase in voltage-gated Ca^2+^ currents, and their potential redistribution to central or peripheral terminals, contributing to inflammation-induced increases in afferent input (Neubert et al., 2000; Bilici et al., 2001; Lu et al., 2010; Takasusuki and Yaksh, 2011). In addition, an increased expression of the alpha2delta auxiliary subunit of VGCCs was observed within the ipsilateral dorsal horn of MIA-(monosodium iodoacetate) induced arthritic rats (Rahman et al., 2009) and the alpha2delta ligand, gabapentin, reduced modalities of hyperalgesia in two different models of knee arthritis (Lu and Westlund, 1999; Vonsy et al., 2008). Further, a subset of OA patients also exhibit nerve injury-like pain and the licensed drugs gabapentin and pregabalin, that modulate VGCC activity, have proven analgesic efficacy for neuropathic pain treatment (Hochman et al., 2011; Ohtori et al., 2012; Roubille et al., 2014). Taken together, these studies suggest that inhibiting VGCCs, in order to reduce the synaptic transmission of the pain signal, is a promising avenue for the treatment of OA pain.

The N-type (Ca_v_2.2) is of particular interest for chronic pain treatment. These channels are located both pre- and post-synaptically on spinal central afferent terminals and second-order neurons, and are crucial for neurotransmitter release, such as calcitonin gene-related peptide (CGRP), substance P (SP), and glutamate and, hence, pain transduction within the CNS (Lee, 2013). The potential of targeting this point of nociceptive convergence was demonstrated by studies showing that selective conotoxins prevented the onset of hyperalgesia or allodynia, and transgenic mice lacking the Ca_v_2.2 gene displayed an altered pain behavioral phenotype (Kim et al., 2001; Saegusa et al., 2001; Scott et al., 2002). Consequently, the selective Ca_v_2.2 blocker, ziconitide (Prialt™), was successfully licensed for the treatment of intractable chronic pain. However ziconitide is of limited use due to a narrow therapeutic window and must be given intrathecally to prevent systemic side effects. Newer therapies, derived and refined from the great promise of N-type blockers such as Ziconotide, with greater therapeutic ratios and more favorable routes of administration could dramatically improve chronic pain treatment. To this end, an orally available, state-dependent Ca_v_2 channel blocker, N-triazole oxindole (TROX-1) was recently developed (Abbadie et al., 2010; Swensen et al., 2012). TROX-1 inhibits all three members of the Ca_v_2 subfamily with equal potency, but, its analgesic activity was quashed in Ca_v_2.2-deficient mice and side effects normally caused by block of Ca_v_2.1 and 2.2 channels were not present, suggesting that TROX-1 efficacy is derived primarily by block of Ca_v_2.2 channels (Abbadie et al., 2010). Importantly, it appears to have an improved therapeutic window compared to ziconitide (Swensen et al., 2012). Notably, TROX-1 significantly reversed mechanical allodynia and hind-limb weight-bearing asymmetry in a rat model of knee OA (Abbadie et al., 2010), suggesting that Ca_v_2.2 channel activity also has an important role in mediating OA pain. Therefore, the aim of this study was to compare the effects of TROX-1 on spinal electrophysiological measures of nociception in a rat model of MIA-induced OA pain. *In vivo* electrophysiology allows for spinal nociceptive processing and central sensitisation to be studied experimentally and provides information on efficacy against suprathreshold responses, which are likely to equate to high levels of pain transmission as reported by patients, therefore adding to behavioral data where the analgesic effect of drugs on threshold responses are generally measured.

## Experimental procedures

A total of 23 Sprague–Dawley rats (Central Biological Services, University College London, UK) were employed for this study. All experimental procedures were licensed by local laws (UK Animals (Scientific Procedures) Act 1986 and the European Communities Council Directive of 24 November 1986 (86/609/EEC)) and followed the guidelines under the International Association for the Study of Pain (Zimmermann, 1983).

### Induction of OA

OA was induced as previously described (Rahman and Dickenson, 2015). Briefly, isofluorane (2% v/v delivered in a 3:2 ratio of nitrous oxide and oxygen) anesthetized male Sprague–Dawley rats (130–150 g) received an intra-articular injection of 2 mg monosodium iodoacetate in 25 μl of 0.9% saline through the infrapatellar ligament of the knee. Sham animals were injected with sterile 0.9% saline only. Following injection animals were allowed to recover and then re-housed in cages under a 12-h alternating light/dark cycle with *ad libitum* access to food and water.

### Behavioral assessment of OA pain

Animals were habituated to the test environment for at least 30 minutes prior to model induction and behavioral testing (carried out on day 7 and day 14 post model induction).

Behavioral assessment of OA pain was carried out as previously described (Rahman and Dickenson, 2015). Briefly, hind limb weight bearing was measured using an incapacitance tester (Linton Instruments, Norfolk, UK) and the weight bearing on the ipsilateral hind limb to knee injection is presented as a percentage of the total weight borne on both hind limbs. Tactile hypersensitivity of the ipsi and contralateral hind paw was tested with von Frey filaments (vFs) (Touch-test TM, North Coast Medical Inc., San Jose, CA, USA) using the “up-down method” (Chaplan et al., 1994), starting with 2.0 g then ranging from 0.4 g to 15 g. Data are presented as mean 50% paw withdrawal threshold (PWT) for each group ± S.E.M. (standard error of the mean).

### *In vivo* electrophysiology

Two weeks after MIA or sham injection *in vivo* electrophysiological studies were performed as previously described (Rahman and Dickenson, 2014). Briefly, animals were initially anesthetized with isofluroane (4% v/v) delivered in a 3:2 ratio of nitrous oxide and oxygen. A laminectomy, under isofluroane anesthesia of 2–3% was then performed to expose the L4–5 segments of the spinal cord. Anesthetic levels of 1.5–1.7% were maintained for the duration of the experiment. Extracellular recordings of the evoked activity of lamina V–VI dorsal horn neurons to electrical and natural mechanical and thermal stimulation of the ipsilateral hind paw were made using parylene-coated tungsten electrodes (A-M Systems, Sequim, WA, USA). All the neurones recorded in this study were of wide dynamic range (WDR) since they all responded to both light touch and noxious inputs (pinch and noxious heat); further all neurones responded to natural stimuli in a graded manner with coding of increasing intensity.

The evoked response to a train of 16 transcutaneous electrical stimuli (2-ms wide pulses, 0.5 Hz, applied at three times the threshold current for C-fiber activation of the dorsal horn cell) was delivered via stimulating needles inserted into the peripheral receptive filed. The neuronal responses evoked by Aβ- (0–20 ms), Aδ- (20–90 ms) and C-fibers (90–350 ms) were separated and quantified on the basis of latency. Responses occurring after the C-fiber latency band were taken to be the post-discharge of the cell (350–800 ms). The center of the peripheral receptive field was then stimulated using mechanical punctate and thermal stimuli (vFs, 2, 8, 26 and 60 g and heat, applied with a constant water jet, 40, 45 and 48 °C). All natural stimuli were applied for a period of 10-s per stimulus. Data were captured and analyzed by a CED 1401 interface coupled to a Pentium computer with Spike 2 software (Cambridge Electronic Design; PSTH and rate functions). The data for the von Frey forces are expressed on a linear scale since the aim was to investigate drug effects on different intensities of stimuli.

Pharmacological assessment was carried out on one neuron only per animal. The testing procedure was carried out every twenty minutes and consisted of a train of electrical stimuli followed by natural stimuli as described above. Following three consecutive stable control trials (<10% variation for the C-fiber-evoked response, and <20% variation for all other parameters) neuronal responses were averaged to give the pre-drug control values. TROX-1 ((3R)-5-(3-chloro-4-fluorophenyl)-3-methyl-3-(pyrimidin-5-ylmethyl)-1-(1H-1,2,4-triazol-3-yl)-1,3-dihydro-2H-indol-2-one)(Synthesized in house; Grünenthal GmbH, Germany), diluted in 0.9% saline solution containing in 5% DMSO (dimethylsulfoxide) and 10% cremophor, was then administered systemically via subcutaneous injection (20 mg/kg) or via topical spinal application (0.1 and 1 μg/50 μl), the vehicle for spinally applied drug was diluted to <1% cremophor and <1% DMSO. The effect of each dose was followed for an hour, with tests carried out at 10, 30 and 50 min before the next dose (spinal administration only) was applied cumulatively. A trend for the greatest effect was seen at either the 10- or 30-min time point (for both routes). Using this protocol the evoked responses are stable for many hours, the lack of drug effect in the sham group evidences this stability.

The vehicle was used for spinal and systemic dosing of TROX-1. Any effect of the vehicle on the observed neuronal effects can be excluded since the drug had no effect in sham animals on any measure by either route. In the MIA animals where the drug was effective, the effects seen were modality selective. Further, a previous study by our group, albeit in a rat model of spinal nerve-induced hypersensitivity, found no effect of the vehicle on behavioral measures of pain after systemic or spinal routes of administration in SNL rats (Patel et al., 2015) nor on electrophysiological recordings of evoked neuronal activity in naïve rats after systemic dosing with the vehicle (unpublished data). Additionally, others have shown that 10% (Menendez et al., 1999) and 50% DMSO (Peng et al., 1997) did not change spinal neuronal activity, and even greater concentrations of intrathecal DMSO did not affect the behavioral response to formalin injection (Lozano-Cuenca et al., 2005). Taken together, we concluded that the vehicle alone was highly unlikely to have had an effect alone, and was not the cause of the inhibitory effects of TROX-1 seen in the present paper. For these reasons vehicle experiments were not undertaken.

### Statistical analysis

All statistical tests were performed on raw data using GraphPad Prism 5 (GraphPad software, USA). Behavioral data were analyzed using the Mann–Whitney test. For *in vivo* electrophysiology measures, statistical significance was tested using either a paired or unpaired Student’s *t*-test to compare two groups of data and a one-way or two-way repeated-measures (RM) analysis of variance (ANOVA), followed by Bonferroni-corrected paired *t*-tests when simultaneously comparing more than two groups of data. For the *in vivo* pharmacological studies the maximal effect, compared with pre-drug baseline control, for each drug dose was selected; this varied and was seen at all the time points tested i.e. 10, 30 and 50 min post drug administration. However, in most cases the maximal change in response was observed at 10 or 30 min post drug application. Drug effects were then expressed as the mean maximal effect of the pre-drug control for each dose. A two-way ANOVA with repeated measures (RM ANOVA) followed by a Bonferroni test for multiple comparisons was used to analyze drug effect on the mechanical- and thermal-evoked neuronal responses. Drug effect on the electrical-evoked responses was assessed using a one-way RM ANOVA followed by Bonferroni test for multiple comparisons after spinal TROX-1 or a paired Student’s *t*-test after systemic TROX-1 administration. Values of *p* < 0.05 were considered significant.

## Results

### MIA-induced pain behavior

**Fig. 1:**
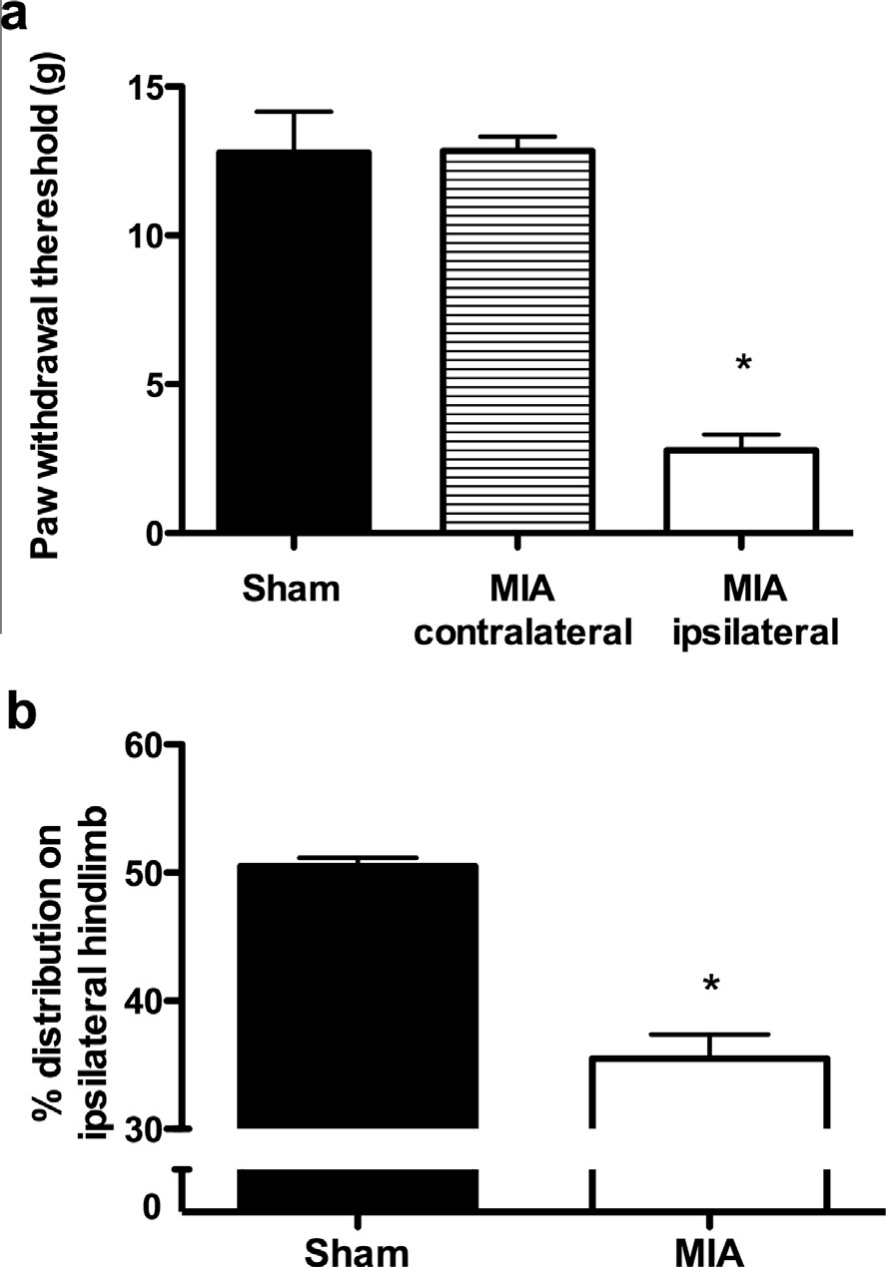
Behavioral assessment at day 14 after MIA (*n* = 12) or saline injection (*n* = 11). Intra-articular injection of MIA injection produced a significant reduction in the paw withdrawal threshold to mechanical punctate stimulation (a) and a decrease in the amount of weight borne on the hind limb ipsilateral to injection (b) compared with the saline-injected sham controls. ^∗^*p* < 0.05 compared with shams, Mann–Whitney test. Values are mean ± SEM.

Intra-articular injection of MIA produced a significant decrease in hind PWT to mechanical stimulation compared with the contralateral hind paw in MIA rats and sham control (Fig. 1a). The behavioral hypersensitive response to mechanical stimulation of the hind paw ipsilateral to MIA injection is indicative of secondary allodynia and central sensitization. A significant reduction in weight bearing of the ipsilateral hind limb in MIA-injected rats was seen compared with the sham control group (Fig. 1b) indicative of enhanced discomfort evoked by weight on the limb in the MIA group.

#### *In vivo* electrophysiology – evoked responses of dorsal horn neurones

The effect of TROX-1, delivered via spinal or systemic route, was assessed upon the evoked responses of deep dorsal horn neurones to electrical and natural mechanical and thermal stimulation of their peripheral receptive field. A total of 23 WDR neurones were recorded. The mean threshold for C-fiber activation was similar between groups as was the mean depth of the neurones, which corresponded with lamina V–VI of the dorsal horn (Table 1). Comparison of the average baseline pre-drug responses for MIA and shams revealed a significantly greater Aδ- and C-fiber-evoked response, and an enhanced Input measure of neuronal excitability in the MIA group. A significantly greater response evoked by mechanical punctate stimulation was seen in the MIA group (*F*_1,3_ = 21.13 *P *< 0.0001) (Table 1), but all other baseline neuronal responses were not significantly different between groups. However, it should be noted that this study was not powered to compare baseline neuronal responses between MIA and sham groups, for this reason any differences in the average baseline neuronal responses were not further analyzed or emphasized. Although spontaneous and enhanced evoked activity of joint nociceptors and dorsal horn neurones has been previously reported (Schuelert and McDougall, 2006, 2008; McDougall et al., 2009; Rahman et al., 2009; Schuelert and McDougall, 2009; Sagar et al., 2010; Kelly et al., 2012; Bullock et al., 2014), as has sensitisation of spinal nociceptive withdrawal reflexes (Kelly et al., 2013) indicating that the MIA model is associated with peripheral and central hyperexcitability.

#### Spinal administration of TROX-1 significantly inhibits the mechanical and thermal-evoked responses of dorsal horn neurones in the MIA group only

**Fig. 2:**
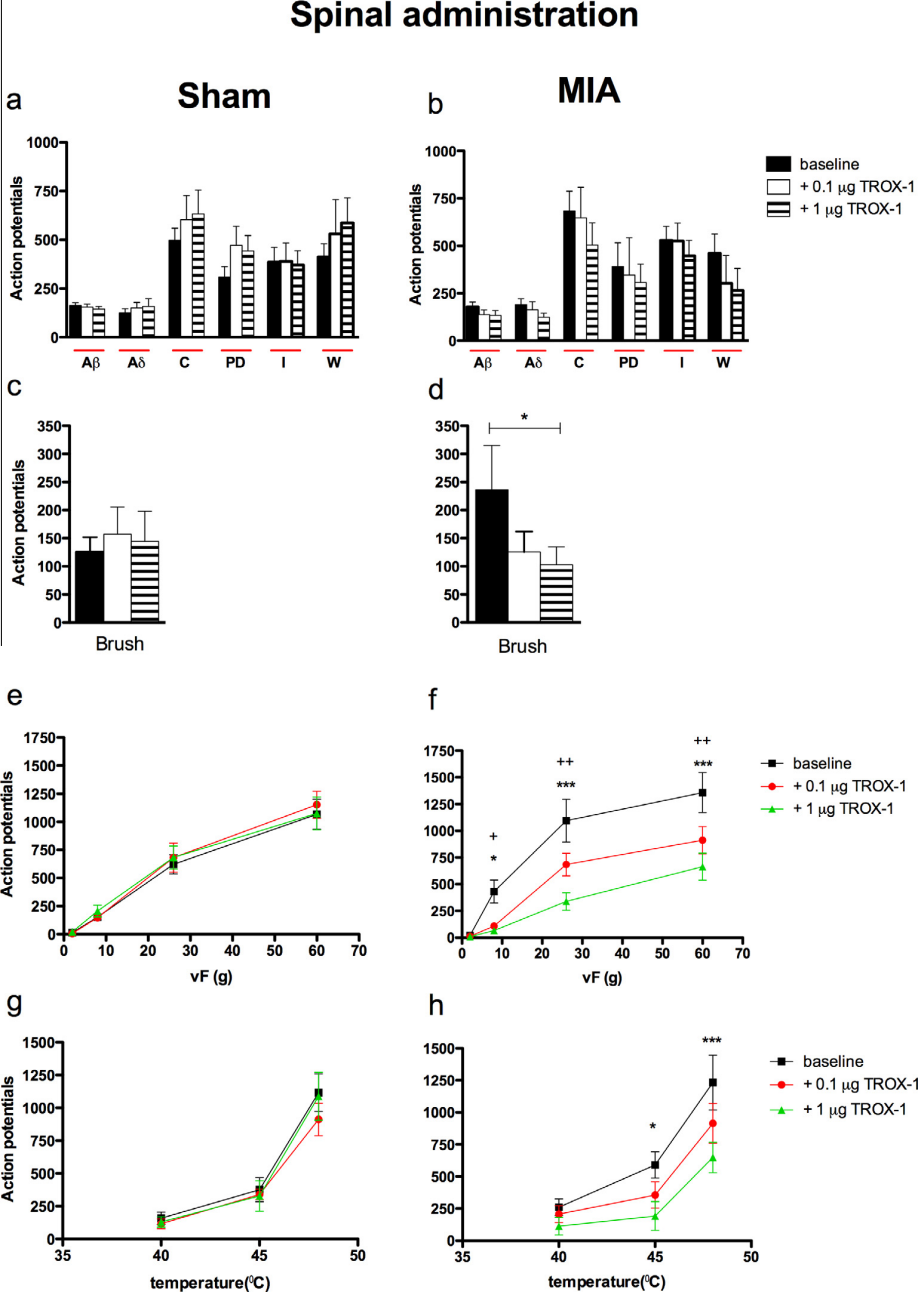
TROX-1 significantly reduced the dynamic brush, mechanical punctate and thermal-evoked neuronal responses in the MIA group. Following stable baseline recordings, OA or sham rats were dosed spinally with 0.1 μg, and 1 μg TROX-1 in a volume of 50 μl. The evoked neuronal response to electrical (a, b), dynamic brush (c, d), mechanical punctate (e, f) and thermal stimulation (g, h) of the peripheral receptive field in sham (*n* = 6, left panel) and MIA (*n* = 6, right panel) rats were recorded. Asterisks and bars denote statistically significant main effect (one-way RM ANOVA). (+) denotes significant difference between baseline and 0.1 μg:^+^*P* < 0.05, ^++^*P* < 0.01. (^∗^) denotes significant difference between baseline and 1 μg TROX-1: ^∗^*P* < 0.05, ^∗∗^*P* < 0.01, ^∗∗∗^*P* < 0.001 (two-way RM ANOVA with Bonferroni post-hoc test). All data are presented as mean ± SEM. PD = post-discharge, I = Input and W = wind up.

Following the characterization of single WDR neurons, the cumulative effects of 0.1 μg and 1 μg TROX-1 applied directly onto to the spinal cord were examined. Spinal TROX-1 did not have any significant effect on any of the electrical-evoked neuronal responses in either the sham or MIA groups. In contrast, the neuronal responses evoked by natural stimulation of the peripheral receptive field in the MIA group were sensitive to the inhibitory effect of TROX-1. Spinal application of cumulative doses of TROX-1 inhibited neuronal responses evoked by dynamic brush stimulation of the peripheral receptive field (*F*_2,5_ = 4.237 *P *= 0.046; Fig. 2d) and mechanical punctate stimuli, (*F*_2,3_ = 30.5 *P *< 0.0001; Fig. 2f) seen as significant decreases in evoked response to stimulation vF 8, 26 and 60 g. A significant inhibition of the evoked neuronal response to noxious heat stimulation after spinal TROX-1 application was also only observed in the MIA group (*F*_2,2_ = 12.84 *P *< 0.0001; Fig. 2h) seen as significant decreases in evoked response to stimulation at 45 °C and 48 °C, whereas the response evoked by innocuous heat stimulation (40 °C) of the hind paw was unaffected by the drug (Fig. 2h). In comparison, TROX-1 had no significant effect on any of the evoked neuronal responses in the sham control group. (Fig. 2d, e, g).

#### Systemic administration of TROX-1 significantly inhibits the mechanical evoked responses of dorsal horn neurones in the MIA group only

**Fig. 3:**
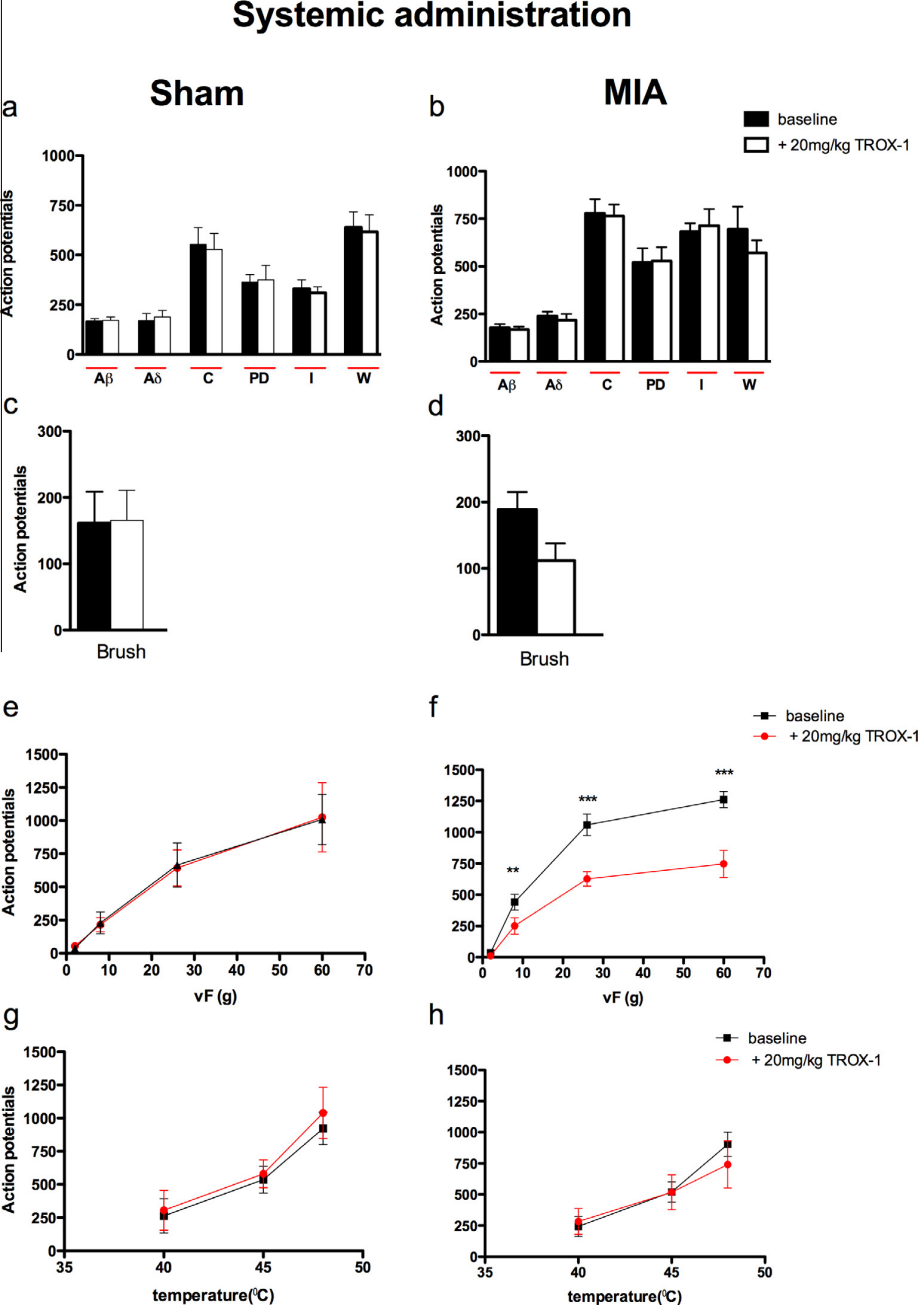
TROX-1 significantly reduced the dynamic brush and mechanical punctate-evoked neuronal responses in the MIA group. Following stable baseline recordings, OA or sham rats were administered a single does of 20 mg/kg TROX-1. The evoked neuronal response to electrical (a, b), dynamic brush (c, d), mechanical punctate (e, f) and thermal stimulation (g, h) of the peripheral receptive field in sham (*n* = 6, left panel) and MIA (*n* = 5, right panel) rats. Asterisk (^∗^) denotes significant difference between baseline and 20 mg/kg TROX-1: ^∗∗^*P* < 0.01, ^∗∗∗^*P* < 0.001 (two-way RM ANOVA with Bonferroni post-hoc test). All data are presented as mean ± SEM. PD = post-discharge, I = Input and W = wind up.

Systemic administration of TROX-1 (20 mg/kg) mirrored some of the effects seen with spinal administration of the drug. The evoked neuronal response to mechanical punctate stimulation was significantly reduced in the MIA group (*F*_1,3_ = 128 *P* ⩽ 0.0001; Fig. 3f) seen as significant decreases in evoked response to stimulation with vF 8, 26 and 60 g. Although a reduction in the evoked neuronal response to brush stimulation was seen in the MIA group with systemic TROX-1, overall this did not reach significance (Fig. 3d). No significant effect was seen with the drug on any of the electrical or thermal-evoked neuronal response in the MIA group (Fig. 3b, h) or on any neuronal measure in the sham group (Fig. 3a, c, e, f).

## Discussion

OA is a progressive and degenerative disease of the whole joint. It is characterized by destruction and degradation of the articular cartilage, synovial lining, connective tissues and subchondral bone remodeling (Vincent and Watt, 2014). In this study we injected the glycolysis inhibitor, MIA (2 mg), into the knee joint, which causes chondrocyte death (Grossin et al., 2004) through an imbalance of anabolic and catabolic pathways, a phenomenon thought to represent a causative factor of OA (Guingamp et al., 1997; Goldring, 2000). Further, the rapid and progressive death of chondrocytes produces cartilage degeneration and perturbations of the subchondral bone, consistent with the clinical histopathology of OA and associated pain (Guingamp et al., 1997; Janusz et al., 2001; Kobayashi et al., 2003). Thus the MIA model has, so far, proved useful for the understanding of osteoarthritic pain mechanisms (Zhang et al., 2013). In the present study joint pathology was not assessed, however, MIA injection produced hypersensitivity to mechanical stimulation of the ipsilateral hind paw and a decrease in hind limb weight bearing of the injected side, confirming pain development and in addition, we have previously observed loss of articular cartilage using this dose of MIA in rats at day 14 post injection (Thakur et al., 2012) as have others (Fernihough et al., 2004; Pomonis et al., 2005; Im et al., 2010), therefore it is highly likely that the animals used in the present study developed OA of the knee and supports the relevance of this model of MIA-induced arthritis for studying OA pain mechanisms (Vincent et al., 2012; Malfait et al., 2013).

Pain symptoms in knee OA patients are largely located within the area surrounding the affected joint, however many OA patients also exhibit areas of referred pain and tenderness, which correlates with the hypersensitivity of the ipsilateral hind paw in MIA rats, and implicates mechanisms of central sensitisation contributing to the pain experience (Farrell et al., 2000; Bajaj et al., 2001; Gwilym et al., 2009; Graven-Nielsen and Arendt-Nielsen, 2010; Aranda-Villalobos et al., 2013) Indeed, experimental human studies in OA patients suggest that central sensitization is an important contributor to their pain and a direct link between sensitization levels in referred areas and clinical pain intensity experienced by OA patients was shown (Arendt-Nielsen et al., 2010). We have recorded the electrophysiological activity of deep dorsal horn WDR neurones, which has been shown to correlate with the intensity and temporal aspects of pain in animals and human subjects (Coghill et al., 1993a,b; Sikandar et al., 2013), therefore the data presented here provide for an electrophysiological correlate for the spread of sensitisation seen in OA patients and allow for spinal nociceptive mechanisms to be studied experimentally.

Spinal and systemic administration of TROX-1 reduced the evoked response to innocuous mechanical stimulation with dynamic brush and vF 8 g in the MIA group, although the effect on brush-evoked responses did not quite reach significance after systemic administration. Nonetheless, these electrophysiological data suggest that TROX-1 would attenuate nociceptive behaviors evoked by low-threshold mechano-stimulation of the hind paw, and correlates with the reversal of tactile allodynia previously reported in a rat model of OA (Abbadie et al., 2010). Suprathreshold mechanical-(vF 26 and 60 g) evoked neuronal responses in the MIA group were also sensitive to the inhibitory effects of spinal and systemic TROX-1 suggesting that the drug would also attenuate mechanical hyperalgesia and therefore may have analgesic efficacy against the more severe pains experienced by some patients.

Spinal administration of TROX-1 attenuated a range of mechanical and thermal-evoked responses in the MIA group indicating that a key site of action of the drug is on VGCCs located on central terminals of primary afferents and/or second-order neuronal networks and possibly DRG. In comparison, systemic administration of the drug was only effective at inhibiting mechanical punctate-evoked neuronal responses, suggesting that mechano-evoked neuronal responses are more sensitive to the inhibitory actions of TROX-1, which may include antagonism of Ca_v_2.2 channels located on peripheral terminals of joint afferents (Just and Heppelmann, 2003) as well as those on central terminals by the systemic route. The inhibition of both thermal and mechanical responses only by spinal application may indicate high local concentrations are able to attenuate both modalities but these are not reached after systemic doses. What is clear is that regardless of the route of administration, TROX-1 was only effective in the arthritic condition. This “functional selectivity” may be due to its state (biased toward open/inactivated channel conformation) and use-dependent activity (Abbadie et al., 2010; Patel et al., 2015), conditions which are more likely in the arthritic state, compared with the sham group, as an increased incidence of spontaneous activity and enhanced responsiveness of joint nociceptors and dorsal horn neurones have been reported in models of OA (Schuelert and McDougall, 2006, 2008; McDougall et al., 2009; Rahman et al., 2009; Schuelert and McDougall, 2009; Sagar et al., 2010; Kelly et al., 2012; Bullock et al., 2014), and we have observed comparatively greater responses of some neuronal measures in the MIA group in the present study (Table 1), which may, in part, underlie the pathophysiological selectivity of TROX-1. Importantly this suggests that, if used in patients, TROX-1 would attenuate abnormal pathophysiological transmission but not normal physiological transmission i.e. acute pain. In line with this hypothesis evidence from studies using Ca_v_2.2 (−/−) mice and ω-conotoxins also suggest that blocking Ca_v_2.2 activity would be more effective against pathological pain compared with acute pain (Sluka, 1997, 1998; Hatakeyama et al., 2001; Kim et al., 2001; Saegusa et al., 2001; Scott et al., 2002).

The MIA-dependent antinociceptive effect of TROX-1 could also reflect an increased expression of Ca_v_2.2 channels within the spinal cord. An increase in Ca_v_2.2 density and a greater functional role within the dorsal horn, ipsilateral to injury, has been demonstrated in models of chronic inflammation and nerve injury (Matthews and Dickenson, 2001; Abbadie et al., 2010; Lu et al., 2010). It is not known if a similar increase in Ca_v_2.2 channels occurs in the MIA model of OA specifically. However, we have previously seen a significant increase in expression levels of the alpha-2delta auxillary subunit of VGCCs within the dorsal horn of MIA rats (Rahman et al., 2009) which may, at least in part, underlie the MIA-selective antinociceptive effect of TROX-1. Interestingly, a recent study demonstrated a link between spinal N-type channel activity, behavioral hypersensitivity and overexpression of VGCC- alpha-2delta overexpression (Chang et al., 2015).

Noxious thermal-evoked neuronal responses in the MIA group were sensitive to the inhibitory effect of TROX-1 after spinal administration but not systemic administration, suggesting that central spinal Ca_v_2.2 channels play an important role in heat hyperalgesia, consistent with the observed anti-nociceptive effects of TROX-1 in inflammation-induced heat hyperalgesia in mice (Abbadie et al., 2010). Surprisingly, we did not observe a similar inhibitory effect on neuronal responses after systemic TROX-1; this may be due to the lower dose, (20 mg/kg), used in our study.

The electrical-evoked neuronal responses were resistant to the inhibitory effect of spinal or systemic administration of TROX-1 this is in marked contrast to its inhibitory effects on the natural-evoked neuronal responses in the MIA group. This could be because the barrage of activity induced by the train of 16 electrical stimuli is maybe too great for the drug at the doses given to elicit any significant effect. Alternatively the two forms of evoked activity may result in a comparatively different proportion of channels entering the open/ inactivated conformational state. We have previously observed enhanced neuronal after discharge (prolonged depolarisations after removal of the stimulus) in MIA rats following mechanical and thermal stimuli applied for 10 s (Rahman et al., 2009). The proportion of channels entering an inactivated state increases with prolonged stimulus (Freeze et al., 2006) therefore this mechanism may enable the state-dependent inhibitor TROX-1 to exert its effect on natural stimuli but not rapid electrical stimulus *in vivo*.

There is mounting evidence for a sub-group of OA patients, refractory to traditional analgesics such as NSAIDs and opioids, who exhibit neuropathic signs and symptoms (Hochman et al., 2011; Ohtori et al., 2012; Roubille et al., 2014). The dose of MIA used here (2 mg) produces OA associated with markers of neuropathy (Ivanavicius et al., 2007; Im et al., 2010; Thakur et al., 2012, 2014), therefore the findings from the present study could translate better to OA patients with a neuropathic component underlying their pain. Interestingly, a recent electrophysiological study by our group using TROX-1 in a rat spinal nerve ligation model of neuropathic pain (Patel et al., 2015) mirrored many of the effects of the dug reported here, suggesting that these chronic pain states share certain common pathological mechanisms, likely to be central rather than peripheral.

## Conclusion

TROX-1 given via spinal and systemic routes of administration significantly reduced neuronal measures of nociception in the MIA group only, suggesting plasticity in Ca_v_2 (likely Ca_v_2.2) channels in the arthritic condition. Further, the findings from the present study support the hypothesis that Ca_v_2.2 blockers would be useful for the treatment of OA pain.

## Figures and Tables

**Table 1:** A comparison of the baseline electrophysiological responses of deep dorsal horn WDR neurons in sham and MIA rats. A significantly greater response was observed for some neuronal measures after MIA injection. The electrical-evoked responses are expressed as the mean number of action potentials (APs) ± S.E.M. evoked by a train of 16 electrical stimuli given at three times the threshold for C-fiber-evoked neuronal response. The natural evoked responses are expressed as the mean number of AP ± S.E.M. evoked during the 10-s period the stimulus was applied to the peripheral receptive field. Asterisk (^∗^) denotes significance compared with sham control data, ^∗^*P* < 0.05, ^∗∗^*P* < 0.01, ^∗∗∗^*P* < 0.001 (unpaired Student’s *t*-test for electrical and brush-evoked responses, two-way ANOVA with Bonferroni post test for the natural mechanical and thermal-evoked neuronal responses)

		Sham (*n* = 12)	MIA (*n* = 12)
Electrical-evoked responses	Depth (μm)	804 ± 36	797 ± 41
C-fiber threshold (mA)	1.1 ± 0.1	1.3 ± 0.2
Aβ-fiber-evoked response (AP)	164 ± 10	178 ± 15
Aδ-fiber-evoked response (AP)	146 ± 67	214 ± 20
C-fiber-evoked response (AP)	523 ± 49	732 ± 62^∗^
Post discharge (AP)	336 ± 33	456 ± 72^∗^
Input (AP)	360 ± 45	606 ± 47^∗∗^
Wind-up (AP)	515 ± 60	578 ± 82

Evoked responses to mechanical stimuli	Dynamic brush	142 ± 25	212 ± 40
vF 2 g (AP)	26 ± 7	29 ± 6
vF 8 g (AP)	187 ± 39	437 ± 59^∗^
vF 26 g (AP)	641 ± 84	1077 ± 104^∗∗∗^
vF 60 g (AP)	1041 ± 107	1310 ± 95^∗^

Evoked responses to thermal stimuli	40 °C (AP)	211 ± 67	253 ± 48
45 °C (AP)	456 ± 69	559 ± 65
48 °C (AP)	1019 ± 94	1083 ± 130
